# From waste to waste: iron blast furnace slag for heavy metal ions removal from aqueous system

**DOI:** 10.1007/s11356-022-19834-3

**Published:** 2022-03-31

**Authors:** Sabah M. Abdelbasir, Mohamed A. Abdel Khalek

**Affiliations:** Central Metallurgical Research and Development Institute, P.O. Box 87, Helwan, 11421 Cairo Egypt

**Keywords:** Blast furnace slag, Removal, Adsorption, Heavy metals, Kinetics, Industrial wastewater

## Abstract

Inordinate levels of heavy metals in water sources have long been a matter of concern, posing serious environmental and public health risks. Adsorption, on the other hand, is a viable technique for removing heavy metals from water due to its high efficiency, low cost, and ease of operation. Blast furnace slag (BFS) is considered a cheap sorbent for the get rid of Co^2+^ and Pb^2+^ ions from aqueous media. The nonmodified slag is characterized using X-ray diffraction (XRD), X-ray fluorescence (XRF), N_2_ adsorption–desorption isotherms, energy dispersive X-ray analysis (EDX), scanning electron microscopy (SEM), and zeta potential. The removal of Co^2+^ and Pb^2+^ ions was carried out using batch adsorption experiments from an aqueous medium. The influence of several variables as pH, contact time, adsorbent dose, temperature, and initial ions concentration was considered. The isotherm, kinetic, thermodynamic, and recyclability were also conducted. The maximum uptake capacity for Co^2+^ and Pb^2+^ was 43.8 and 30.2 mg g^−1^ achieved at pH 6 after 60 min contact time. The adsorption kinetics and isotherms of BFS for Co^2+^ and Pb^2+^ fitted well to Avrami and Freundlich models, respectively. The main adsorption mechanism between BFS and the metal ions was ion exchange. The regeneration of the used slag was studied for reuse many cycles. In terms of economics and scalability, nonmodified BFS treatment has great potential as a cost-effective adsorbent that could be used in water pollution treatment.

**Graphical abstract:**

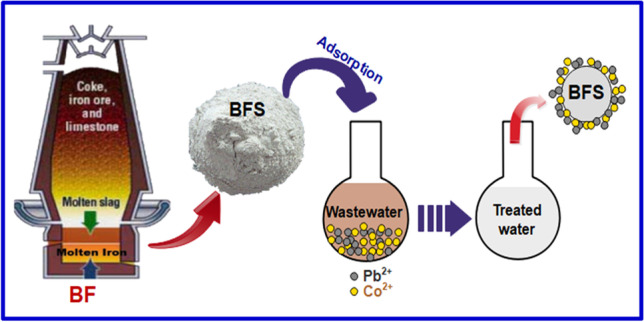

**Supplementary Information:**

The online version contains supplementary material available at 10.1007/s11356-022-19834-3.

## Introduction

Noxious heavy metals generated by a variety of manufacturing practices can cause significant environmental harm if not efficiently eliminated from the waste discharges (Sall et al. 2020). These heavy metals adversely affect human health, the environment, and aquatic systems when they accumulate in living creatures at levels above the permitted limits (Fu and Wang 2011; Gupta et al. 2012; Ihsanullah et al. 2016). Among the heavy metal ions, Pb^2+^ and Co^2+^ represent a greater hazard to human health. Acute Pb^2+^ exposure, for example, can result in newborn brain harm as well as nervous system, kidney, and vascular system disorders (Fathy et al. 2021). Generally, when Pb^2+^ ions compile in living cells, they interact with the proteins’ sulfhydryl group disrupting many biological and metabolic activities (Wang et al. 2016). Cobalt noxiousness can induce asthma symptoms as well as liver, thyroid, and heart problems. At high concentrations, it can also cause genetic changes in living creatures (Jaishankar et al. 2014; Sall et al. 2020; Briffa et al. 2020). Furthermore, these two metal ions are recognized as potential cancer-causing agents, and they have been used as model contaminants for adsorption researches despite their toxicity (Khulbe and Matsuura 2018; Abdelbasir et al. 2021).

Eliminating these metals from polluted water is critical for both human health and environmental conservation. Metals are traditionally precipitated by adding hydroxyl or sulfate agents. Nevertheless, those techniques yield significant quantities of hazardous byproducts and are ineffective for negligible metals concentrations (Bazrafshan et al. 2015). For these waters having low metal concentrations, activated carbon adsorption, ion exchange, and membrane technology are effective management options (Alafif et al. 2019). However, the considerable expense and need for pretreatment are disadvantages of these methods. As a result, a frequently recommended practice for the elimination of heavy metal ions from waste discharges has been the use of economically affordable adsorbents (Saleem et al. 2019). Adsorption process is one of the supreme widely applied methods for wastewater treatment since it is very effective in eliminating pollutants and it is low cost mainly when adding low adsorbent dose (Badawi et al. 2021). There are several sorbent materials that could be utilized for heavy metal adsorption (Abdelbasir et al. 2020; Badawi and Zaher 2021). Such materials comprise, for instance, widely accessible raw materials and wasted industrial byproducts (Nguyen et al. 2018). Natural polymers and zeolites, clay minerals, peat, ash, and slag are the most commonly investigated affordable adsorbents (Carvalho et al. 2011). Notwithstanding its extensive application, the adsorption practice has its own constraints. The most difficult task is the advancement of a sorbent material that is fit for a concurrent and efficient getting rid of contaminants from wastewater at ultra-low levels (Sen Gupta and Bhattacharyya 2014).

Blast furnace slag (BFS) is produced in huge quantities by iron and steel companies, which pose a large major disposal challenge. In 2013, global steel slag output was approximated to be between 170 and 250 million tonnes (Gomes et al. 2018). BFS is a non-metallic output of steel manufacturing. Blast furnaces run at temperatures about 2000 °C and are supplied with regulated mixtures of iron ore (Fe_2_O_3_ + SiO_2_), coke (C), and limestone (CaCO_3_) and the end products are steel and slag (Medina et al. 2020). Even though the majority of the slag has been disposed of as junk, it has found uses in building and soil improvement. Because BFS is retrieved at high temperatures, the metals present are firmly bound to its matrix and do not easily leach, making it environmentally safe (Kanel et al. 2006). Furthermore, BFS has a high uptake capacity for heavy metals due to the existence of Si and Fe oxides and due to its availability and chemical composition, it can be used as an adsorbent for metals (Liu et al. 2010; Beh et al. 2012; Ahmed and Ahmaruzzaman 2016), phosphate (Xiong et al. 2008; Han et al. 2016), and dyes (Xue et al. 2009; Gao et al. 2017). It can be also used as Fenton-like catalytic agents to break down various organic contaminants (Arzate-Salgado et al. 2016; Nasuha et al. 2016; Cheng et al. 2017). However, several minor components present in steel slag tend to concentrate on the slag surface during crystallization, affecting the adsorption to pollutants. Moreover, due to the limited pore structure of BFS, the internal components are unavailable for utilization efficiently, resulting in a limited adsorption efficiency of the slag. Several researchers have employed various activation and/or conversion technologies to improve the adsorbing performance of BFS. Zhan et al. (2019) employed bentonite-steel slag composite powders as an adsorbent to treat acid mine drainage containing Pb^2+^. Chen et al. (2020) prepared acid-modified steel slag as a new type of adsorbent to remove U^6+^ in an aqueous solution. Slag has been converted to calcium silicate hydrate to remove Pb^2+^, Zn^2+^, and Cu^2+^ from wastewater (Yang et al. 2019), and to remove Sr^2+^ and Cs^+^ (Tsutsumi et al. 2014). To adsorb Co^2+^ ions from aqueous solution, it was also converted into Slag-Oxalate composite (Le et al. 2021). NaOH was used to activate BFS, which was then used to remove Ni^2+^ from aqueous solutions (Sundhararasu et al. 2021). The post-grafting method was used to modify BFS with γ-aminopropyltriethoxysilane (APTES) to improve its adsorption performance (Wang et al. 2021).

Most of the previous works were focused fundamentally on BFS modification or conversion to maximize its chemical and economic potentials. So, the target of our work was to look into the use of BFS, as it is without modification or conversion to other material, for the elimination of cobalt and lead ions from wastewater by adsorption. To evaluate its performance as an adsorbent, batch experiments were carried out and different parameters such as the initial concentration, pH, adsorbent dose, contact time, and temperature were considered. The relating adsorption isotherms, kinetics, and thermodynamics were thoroughly studied. The findings of this study will have a major impact on the use of low-cost adsorbents for wastewater treatment, resulting in reduced waste generation.

## Experimental

### Materials

A 5 kg sample of iron slag was obtained as a byproduct from the Egyptian Iron and Steel Co., Tabbin, Egypt (29.80° N and 31.31° E). It was firstly rinsed with pure water for surface impurities removal then dried out at 105 °C for a whole night. It was ground and classified according to particle size. The sample was endured to a size reduction by means of a rotating ball mill with nineteen steel balls weighing 540 g each; the longer the grinding time (almost 2 h), the finer and non-agglomerated the particles became.

Nitrate salts of cobalt [Co(NO_3_)_2_] and lead [Pb(NO_3_)_2_] (pure Sigma-Aldrich and Merck grade) were utilized for the preparation of Co^2+^ and Pb^2+^ stock solutions using ultrapure water. Standard solutions of NaOH and HCl (0.1 mol L^−1^) were applied for pH monitoring.

### Characterization

Chemical composition and full characterization of BFS were determined using different characterization tools (see supplemental file for full details). The chemical composition of the BFS was detected as metal oxides. The concentration of Co^2+^ and Pb^2+^ ions was determined by atomic absorption spectroscopy (GBC-908136 AA, Australia) (Limiju 2017).

### Adsorption experiments

The characterized slag was applied as an adsorbent for Co^2+^ and Pb^2+^ ions. Adsorption experimentations were executed using 0.05 g of slag powder in 30 mL of ions solution of desired concentration, temperature, and pH in a 50 mL round bottom bottle. The bottle was shaken in a water bath at 200 rpm for a certain time. Then, the solid adsorbent is removed using a centrifuge. The residual ions were analyzed by atomic absorption spectrophotometer (AAS).

The amount of adsorbed ions per unit weight of slag, *q* (mg/g), is determined from the following relation (Maged et al. 2020):

1 $$\mathrm A\mathrm d\mathrm s\mathrm o\mathrm r\mathrm p\mathrm t\mathrm i\mathrm o\mathrm n\:\mathrm c\mathrm a\mathrm p\mathrm a\mathrm c\mathrm i\mathrm t\mathrm y\;''q''\;\left(\mathrm{mg}/\mathrm g\right)=\frac{\left(\mathrm{Ci}-\mathrm{Cf}\right)\times\mathrm V}{\mathrm W}$$

The percent removal of ions was estimated from the following relation (Abdelbasir et al. 2021):

2 $$\mathrm{Removal\:efficiency\:\%}=\frac{{C}_{i}- {\mathrm{C}}_{f}}{{C}_{i}}\times 100$$

Applying that: *V* is the solution volume in liter (30 mL = 0.03 L), *W* is the slag dose (g), *C*_*i*_ and *C*_*f*_ are the original and final ions’ concentrations (mg L^−1^).

### Regeneration and desorption study

To lower the expenses of the sorption practice and recover the contaminant removed from the waste effluent, the regeneration of the adsorbent is a necessity. A 0.1 mol L^−1^ HNO_3_ solution was used to study the desorption of the adsorbed ions at 60 °C for 30 min. (1:10 solid–liquid ratio). Solutions were finally separated from the solids by centrifugation followed by filtration. AAS was used to measure the ions concentration preceding to and following desorption experiments. Equation (3) was used to calculate the desorption efficiency:

3 $$R_{\mathit d\mathit e\mathit s}=\frac{D_{\mathit d\mathit e\mathit s}}{A_{\mathit d\mathit e\mathit s}}\times100$$

The amounts of adsorbed and desorbed metal ions are represented by *A*_*ads*_ and *D*_des_, correspondingly. Reusability was achieved by utilizing the regenerated adsorbent in subsequent adsorption experiments and repeating the adsorption–desorption method with the same adsorbent sample.

## Results and discussion

### Slag characterization

**Fig. 1:**
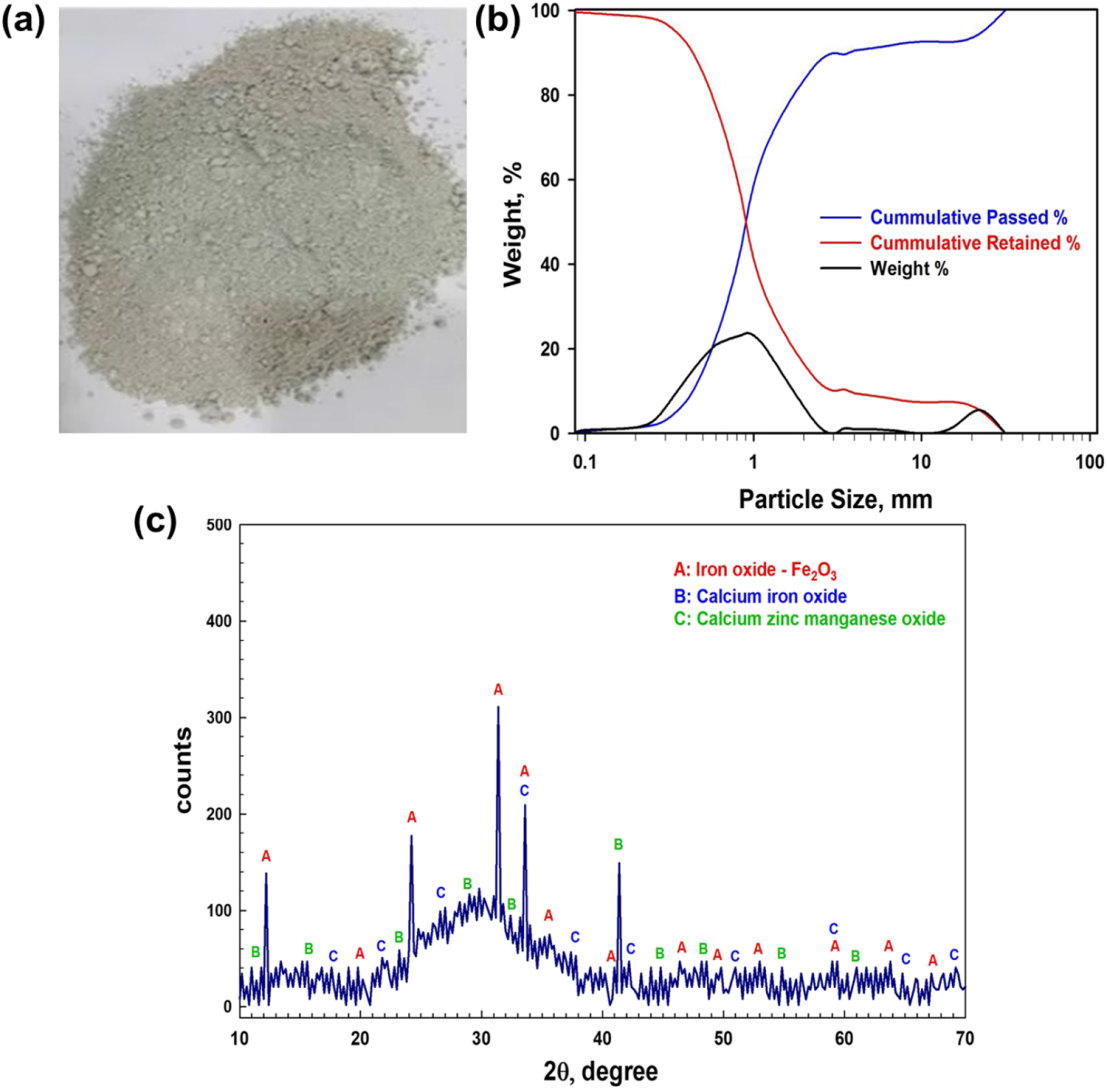
**a** A photo of the ground BFS, **b s**ize analysis of the ground sample, weight, cumulative passed and retained, and **c** XRD pattern of BFS

The X-ray fluorescence (XRF) analysis of BFS was achieved after grinding and the elemental composition is shown in Table 1. The average content of Ca as oxide (CaO) was approximately 46.62%, as the main element of BFS, followed by Si, Ba, Mn, and Mg. The values represent an average duplicate of BFS analysis. Figure 1a shows a photo of the ground BFS.

**Table 1:** Chemical composition of blast furnace slag

Element	Na	Zn	Mg	Si	S	K	Ca	Ti	Mn	Fe	Ba	Others
Wt. % as oxide	0.80	0.14	2.93	23.7	2.02	1.02	46.62	1.08	8.88	0.93	11.15	0.73

The particle size analysis of the slag is shown in Fig. 1b. It is seen that about 50% of the sample has a particle size less than 0.90 mm, while a 90% weight of the sample is less than 0.43 mm. Also, about 80% of the sample has a particle size range of 0.417–1.16 mm. The degree to which finer particles are reduced is widely known to be subordinate to the material type, the mill, and the grinding circumstances (Petrakis and Komnitsas 2019).

Figure 1c depicts the X-ray diffraction (XRD) pattern of BFS. The pattern is quite intricate which is mostly due to the raw material nature. The sample is possibly amorphous glassy having a hump at about 2θ:28°–33° (Mostafa et al. 2001). It is also worth mentioning that quite a few peaks were found indicating the presence of crystal phases in the slag.

**Fig. 2:**
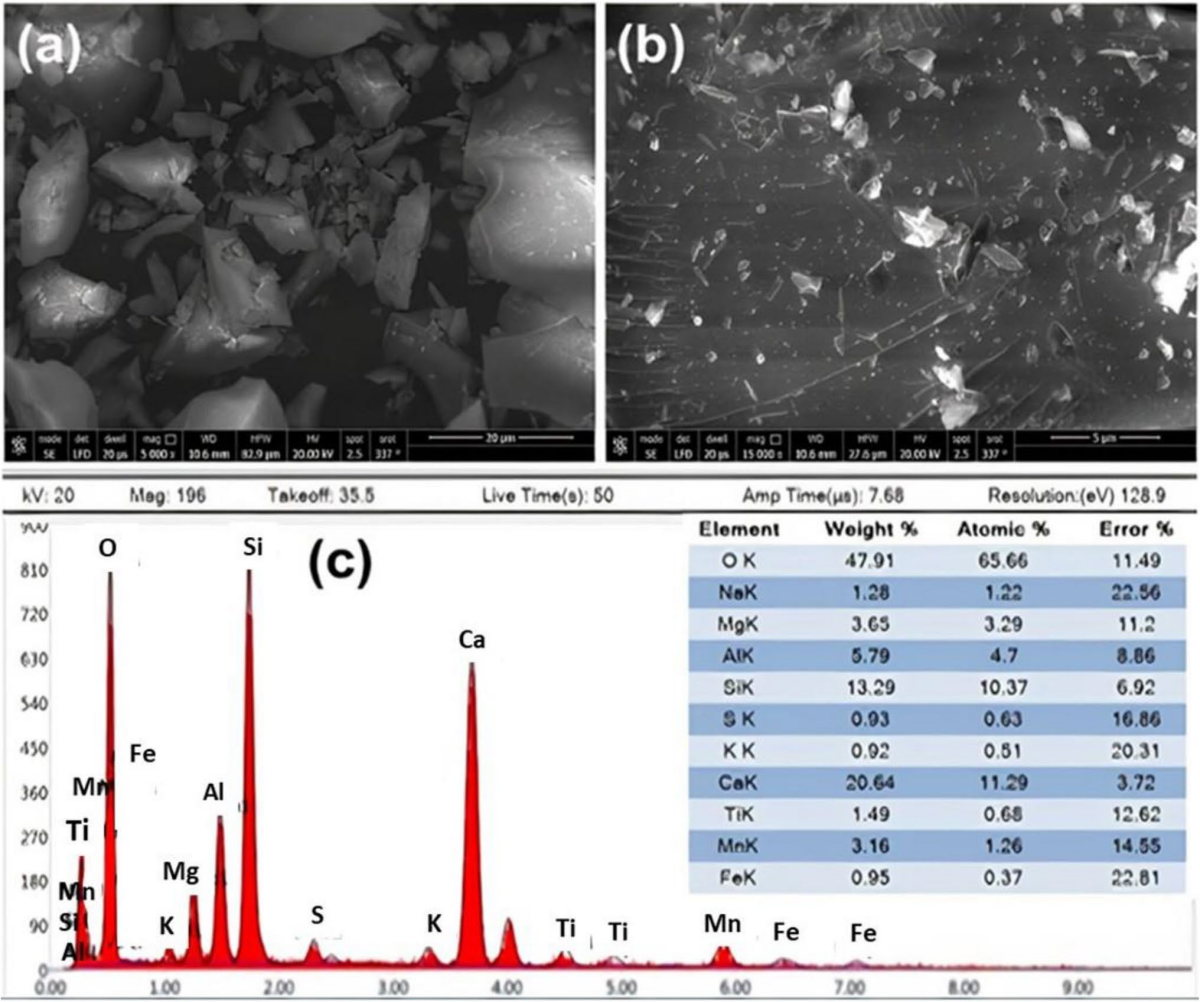
SEM images: **a** 20 μm, **b** 5 μm, and **c** EDX analysis of BFS

SEM and energy-dispersive X-ray (EDX) analyses were utilized to define the size and basic structure of the BFS as displayed in Fig. 2. SEM images revealed the coarse, slack, and porous surface textures of the BFS sample (Fig. 2a and b). Slag particles had become angular in form, with definite asperities and edges evident. Rough surface textures were also a feature of them. The EDX analysis revealed high-intensity peaks for Ca, O, Si, Al, Mg, and other noticeable ones for Na, Ti, and S, in conformity with the analysis and XRD outcomes. Steel slags are known to include oxides, which are produced during the steelmaking process.

CaO is the most common oxide found in steel slags generated during diverse steelmaking processes (Yildirim and Prezzi 2011). In our case, FeO is another oxide generated during some steelmaking practices with a low occurrence. Slag leachates are also frequently found to be extremely basic because of CaO and further basic oxides (Riley and Mayes 2015).

**Fig. 3:**
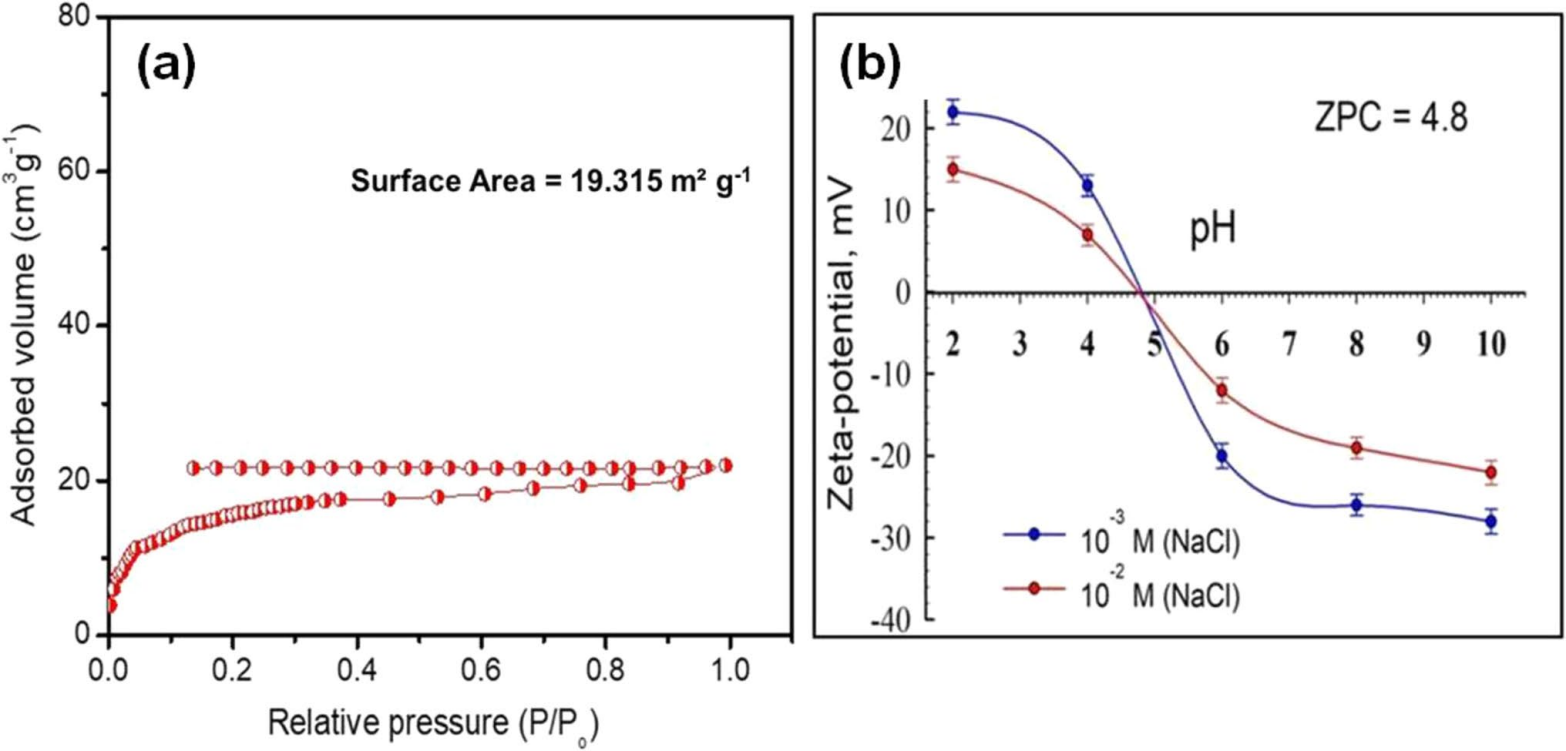
**a** N_2_ adsorption isotherm and **b** zeta potential of the BFS

The surface area and the pore structure of BFS were assessed by the nitrogen isotherms analysis shown in Fig. 3a. As we can see from the figure, the shape of the isotherm is classified as Type II, indicating that the slag with a heterogeneous granular aspect established the slit aperture shaped by the particles’ accumulation (Deng et al. 2020). The specific surface, pore-volume, and average diameter of BFS are revealed in Table S1 in the supplemental file.

Figure 3b shows the surface charge results of the slag in an electrolyte solution. The slag surface charge is clearly pH-dependent, being positively charged at pH less than 4.8, and becoming negatively charged as the solution pH progressed to neutral and basic regions. A pH 4.8 was found to be the isoelectric point (IEP).

### Adsorption experiments

Adsorption batch experimentations were applied to validate the heavy metal removal efficiency using BFS as adsorbent for Pb^2+^ and Co^2+^ ions from the prepared solutions.

#### Impact of pH

**Fig. 4:**
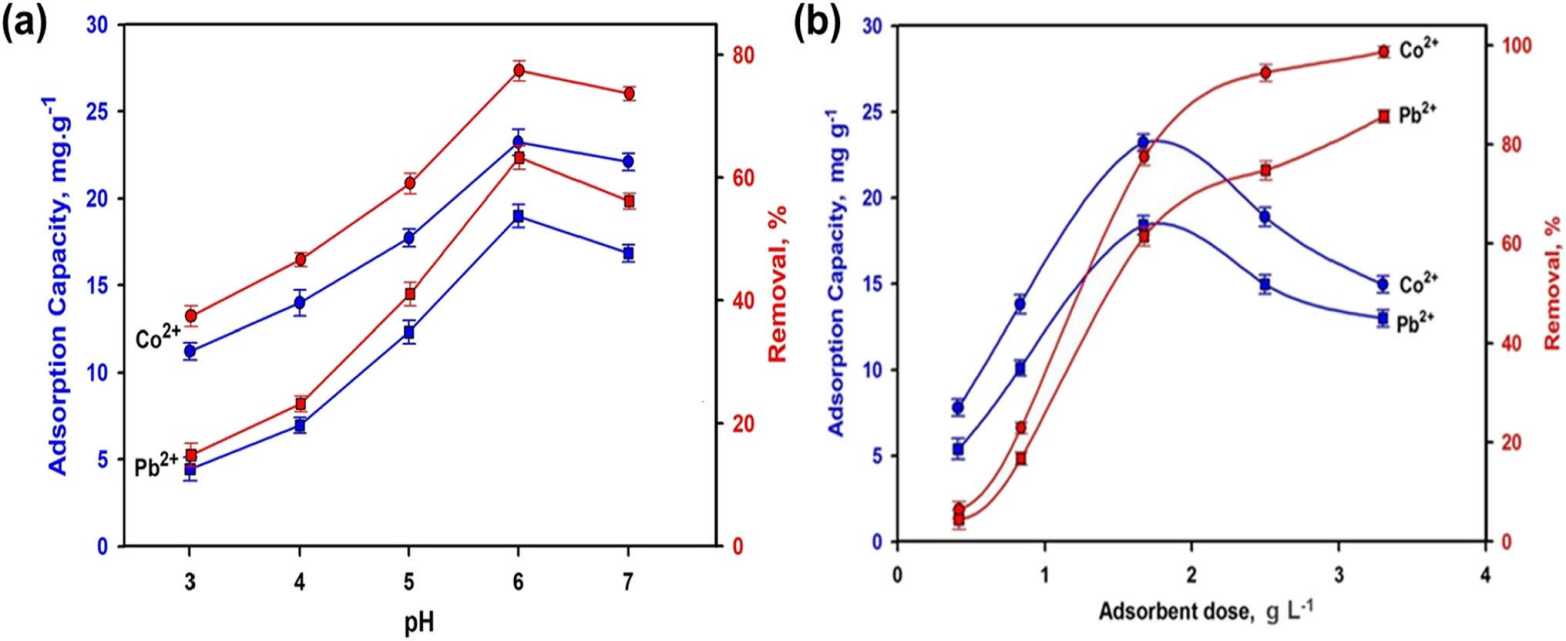
**a** Effect of pH (initial concentration: 50 mg L^−1^, adsorbent doze: 1.67 g L^−1^, contact time: 60 min at room temperature). **b** Effect of slag dose (initial concentration: 50 mg L^−1^, contact time: 60 min at room temperature) on the removal efficiency and adsorption capacity of slag

The sorption procedure is greatly impacted by the medium’s pH. Figure 4a shows the removal efficiency and uptake capacity of different ions from synthetic solution. The maximum sorption capacities and removal efficiency are accomplished at pH 6. Metal ions solubility reduces at alkaline pH, enabling precipitation and complicating sorption (He et al. 2015). The concentration of external H^+^ rose at low pH levels which are considered as competitive in ion exchange (Akhigbe et al. 2015; Kozera-Sucharda et al. 2020). The slag selectivity follows the order of Co^2+^  > Pb^2+^. The charge density (charge/ionic radius), hydration energy, and proportions of the hydrated ions may all be used to predict the solid’s vantage for various ions all through competitive sorption (see Table S2 in the supplementary file). Other factors, such as the geometry and/or orientation of the ions, also influence selectivity. As well, the distribution of the surface charge on slag can change based on its composition and activation. As a result, solution pH has a significant impact on the adsorbent’s functional group activity. The slag surface charge is negative at a pH higher than 4.8 (Dimirkou 2007; Acheampong et al. 2010; Elboughdiri 2020). The distinct ability to adsorb ions is due to the slag’s containment (Wang et al. 2021).

#### Impact of sorbent dosage

Figure 4b validates the action of the slag dose (g L^−1^) on the elimination efficiency and uptake capability of the slag. The amount of added slag to the aqueous solution significantly affects the adsorption process. Intensifying the dose caused an upsurge of the removal efficiency while the adsorption capacity was increased up to 1.67 g L^−1^, and then decreased. Each metal ion is subjected to a larger unit mass of the adsorbent, which has more adsorption sites which ready to attract this ion (Mahmoud et al. 2019).

#### Impact of original metal ion concentrations and sorption isotherms

**Fig. 5:**
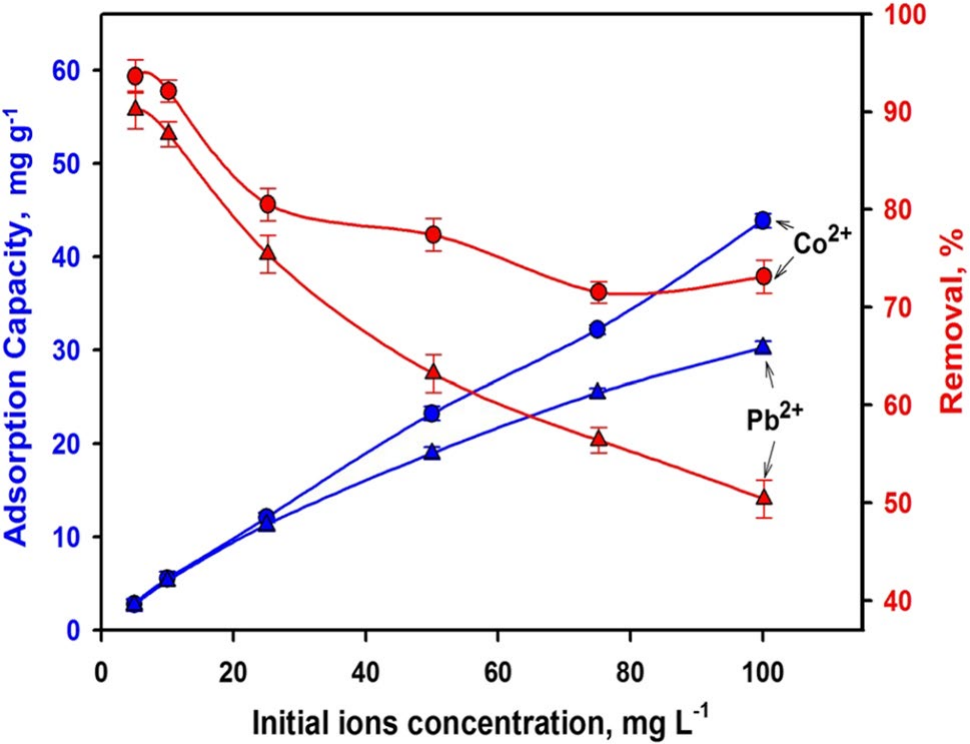
Effect of initial concentration on the removal efficiency and adsorption capacity (adsorbent doze: 1.67 g L^−1^, contact time: 60 min, at solution pH: 6 and room temperature)

Figure 5 exhibits the initial ion concentration’s influence on the removal efficacy and uptake capacity of the slag. The concentration of ions in an aqueous medium significantly affects the adsorption practice (Abdel-Khalek et al. 2017). Increasing the initial ion concentration increased the uptake capacity and removal efficiency. A high starting concentration indicates that more ions are accessible and hence, more ions are sorbed for a fixed sorbent’s amount (Khalek et al. 2019). At a higher initial concentration, the driving forces to conquer the mass transfer barrier for ions’ emigration through the medium to the sorbent solid surface upsurges. Nevertheless, each unit weight of the sorbent is exposed to a greater amount of ions steadily loading the sites until fullness (Mahmoud et al. 2019).

The adsorption isotherms are the best analysis method to describe the sorption behavior (Hałas et al. 2017)**.** Temkin, Langmuir, and Freundlich isotherms were employed to investigate the sorption practice.

#### Temkin isotherm

**Fig. 6:**
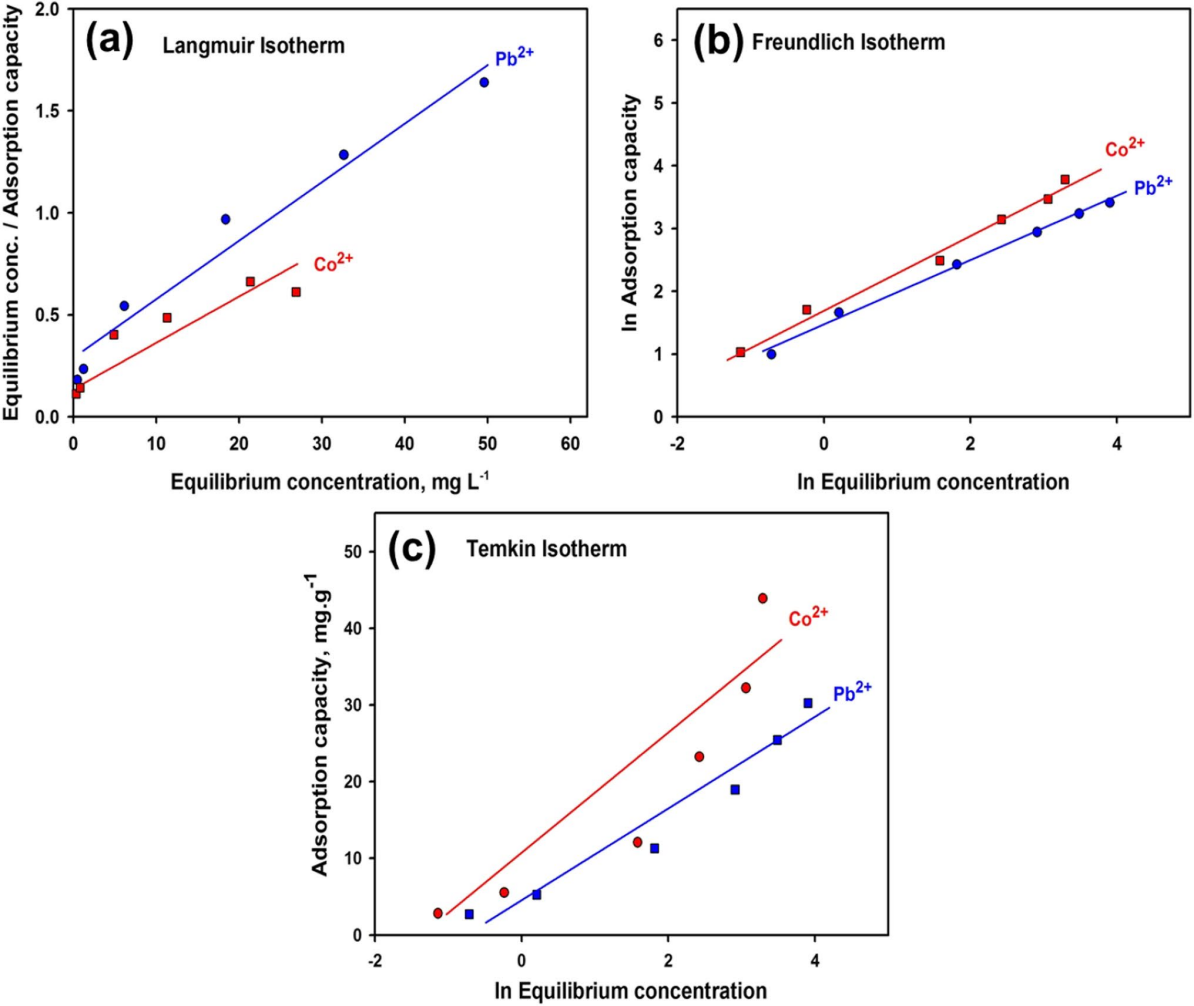
Plotting results according to **a** Langmuir, **b** Freundlich, and **c** Temkin isotherms ((initial concentration: 50 mg L^−1^, contact time: 60 min at room temperature)

According to the Temkin model, the adsorption heat of sorbed species in the layer declines linearly rather than logarithmically as a function of temperature (Ostrovskii 1989; Ho and McKay 1998; Hoslett et al. 2020). Temkin model equation is written as (Tsai and Chen 2013):

4 $${q}_{e}={\mathrm{BlnA}}_{\mathrm{T}}+\mathrm{B ln}{\mathrm{C}}_{\mathrm{e}}$$

where *q*_*e*_ is the adsorbed ions at equilibrium, A_T_ is the equilibrium constant interrelated to the maximal binding energy (L g^−1^), B is the Temkin isotherm constant linked to the heat of adsorption (J/mol), R is the universal gas constant (8.314 J/mol/K), and T is the temperature in kelvin. By plotting q_t_ against ln C_e_, the constants were assessed from the plot intercept and slope (Fig. 6). A_T_ and B values are found in Table 2 (the computed *R*^2^ values are 0.8476 and 0.8914).

**Table 2:** Parameters of Temkin, Langmuir, and Freundlich isotherm models for the slag

Isotherm	Parameter	Pb^2+^	Co^2+^
Temkin	*R*^2^	0.8914	0.8476
	*B*	5.7920	8.1673
	*A*_T_ (L/g)	2.1557	2.5745
	RMSE	9.3838	12.3143
Langmuir	*R*^2^	0.9655	0.8372
	*b* (L/mg)	0.1062	0.0986
	*q*_max_(Cal.)	34.0	52.1
	*q*_max_ (Exp.)	30.2	43.8
	RMSE	2.1254	12.3154
Freundlich	*R*^2^	0.9950	0.9895
	*n*	1.958	1.707
	*K*_f_ (mg/g)	4.293	5.631
	RMSE	0.5492	0.6731

#### Langmuir isotherm

Langmuir model’s equation (Chen et al. 2012) is:

5 $$\frac{{C}_{f}}{{q}_{t}}=\frac{{C}_{f}}{{q}_{\mathrm{max}}}+\frac{1}{{bq}_{\mathrm{max}}}$$

Knowing that C_f_ (mg L^−1^) is the final ions concentration, *q*_*t*_ (mg g^−1^) is the adsorbed ions’ amount at time *t*, *q*_max_ (mg g^−1^) (highest sorption) is monolayer sorption capacity, and *b* (L mg^−1^) is a constant associated with the sorption energy. Langmuir model adopts that the occurrence of sorption at a precise homogeneous surface of the adsorbent where the ions flow through the pores and the apertures of the lattice to replenish the substitutable ions of the sorbent. From Fig. 6 and Table 3, the regression (*R*^2^) value of Langmuir model linear fitting 0.8372 and 0.9655.

**Table 3:** The dimensionless separation factor or equilibrium parameter (R_L_)

Initial conc., mg L^−1^	R_L_	
	Pb^2+^	Co^2+^
5	0.9888	0.9896
10	0.4850	0.5035
25	0.2736	0.2886
50	0.1585	0.1686
75	0.1115	0.1191
100	0.0861	0.0921

#### Freundlich isotherm

This isotherm model (Visa 2016) equation could be written as:

6 $${ln q}_{t}=ln{K}_{f}+\frac{1}{n}ln {C}_{f}$$

where *K*_*f*_ (Freundlich constant, mg/g) is the connotative of the degree of the sorption and *n* is the sorption intensity.

*K*_*f*_ points to the uptake capacity, whereas 1/*n* is a function of the sorption capability (Tsai and Chen 2013; Shehab et al. 2019). If *n* = 1, the barrier between the two phases is unaffected by concentration. If *n* is less than 1, it assigns typical adsorption and if it lies between 1 and 10, a favorable sorption process is nominated (Goldberg 2018). From Table 2, the values of *n* are 1.707 and 1.958 while *R*^2^ values are 0.9895 and 0.995, indicating that the sorption procedure is favorable and of physical character (Abdel-Khalek et al. 2020).

#### The dimensionless equilibrium parameter or separation factor (RL)

It is assigned as:

7 $${\mathrm{R}}_{\mathrm{L}}=\frac{1}{\left(1+{\mathrm{bC}}_{0}\right)}$$

Implying that C_0_ (mg L^−1^) is the initial metal ions’ concentration and *b* is Langmuir’s constant. R_L_ value specifies whether the sorption is advantageous or non-advantageous. If R_L_ values lie between 0 and 1 then, the sorption procedure is auspicious, whereas R_L_ = 1 denotes non-advantageous linear sorption, and R_L_ = 0 signifies non-reversible sorption.

The results showed that the value of R_L_ is almost unity with 5 mg L^−1^ which indicates unfavorable adsorption. While at other concentrations up to 100 mg L^−1^, R_L_ is between 0 and 1 designating advantageous adsorption.

#### Time impact and sorption kinetics

**Fig. 7:**
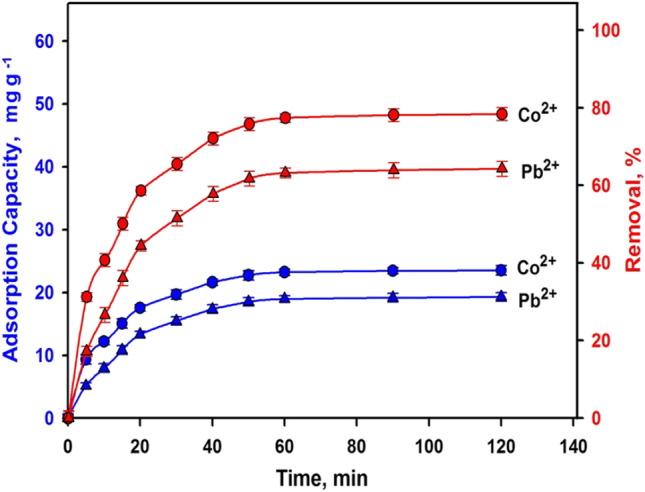
Effect of conditioning time on the removal efficiency and adsorption capacity of slag (initial concentration: 50 mg L^−1^, adsorbent doze: 1.67 g^−1^, solution pH: 6 at room temperature)

To find an appropriate contact time for the adsorption, the uptake capacity and removal efficiency of various ions were evaluated as a function of time (Fig. 7). The equilibrium time was determined to be 60 min. The increased sorption rate at the early 20 min is owing to the accessibility of ions and vacant sorption spots on the slag’s surface. Then, the sorption active spots steadily lessened, and the extent of sorption was assessed by the number of ions transferred from the solution to the sorption active spots. Thus, the sorption increases with time until fullness is reached (Zare et al. 2018; Abdel-Khalek et al. 2020). Also, the ions required more time to penetrate the tiny pores. The higher initial rate implies that the adsorption happens on the exterior surface first, ensued by the interior pores (Nguyen et al. 2018). Furthermore, the larger adsorption amount in the first period demonstrated higher sorption on the exterior surface rather than in the pores (Zare et al. 2016).

The sorption extent of metal ions by the slag was examined via the Lagergren pseudo-first-order and pseudo-second-order models. Also, the Avrami model was used which describes a fractional kinetic order (Lopes et al. 2003; Issaoui et al. 2021). The Lagergren for the pseudo-first-order (PFO) and pseudo-second-order (PSO) models were denoted as shown in the coming equations:

8 $$\begin{array}{cc}\mathrm{PFO}\:\mathrm{model}&\ln\left({\mathrm q}_{\mathrm e}-{\mathrm q}_{\mathrm t}\right)={\mathrm{lnq}}_{\mathrm e}-{\mathrm K}_1\mathrm t\end{array}$$

9 $$\begin{array}{cc}\mathrm{PSO\:model}& \mathrm{t}/{\mathrm{q}}_{\mathrm{t}}=1/{\mathrm{K}}_{2}{{\mathrm{q}}_{\mathrm{e}}}^{2}=\mathrm{t}/{\mathrm{q}}_{\mathrm{e}}\end{array}$$

where k_1_ (min^−1^) and k_2_ (g mg^−1^ min^−1^) are the equilibrium rate constants.

The Avrami model was expressed as follows (He and Duan 2016; Narayanan et al. 2020):

10 $$\ln\left[-\mathit{ln}\left(1-\mathrm\alpha\:\right)\right]=n\;\ln K_{avr}+n\;\ln\;t$$

The model explains a system with a time-dependent rate coefficient. It provides the greatest fitting of metal ion intake on sorbents (Issaoui et al. 2021; Vaghetti et al. 2009). The integral form is where *K* is the Avrami constant, and *n* is a constant interrelated to the sorption mechanism.

11 $${\mathrm{Q}}_{t}={Q}_{m}-{Q}_{m} {e}^{-{K}_{avr}{.t}^{n}}$$

12 $$\frac{{Q}_{t}}{{Q}_{m}}=1-{e}^{-{K}_{avr}{.t}^{n}}$$

The $\frac{{Q}_{t}}{{Q}_{m}}$ is the adsorption fraction “α”. By plotting “$\mathrm{ln}\left[-\mathit{ln}\left(1-\mathrm{\alpha }\right)\right]$” versus “ln t,” the *n* and *K* can be computed from the intercept and slope as follow: the slope equal “*n*” and the intercept equals “*n* ln *K*.”

**Fig. 8:**
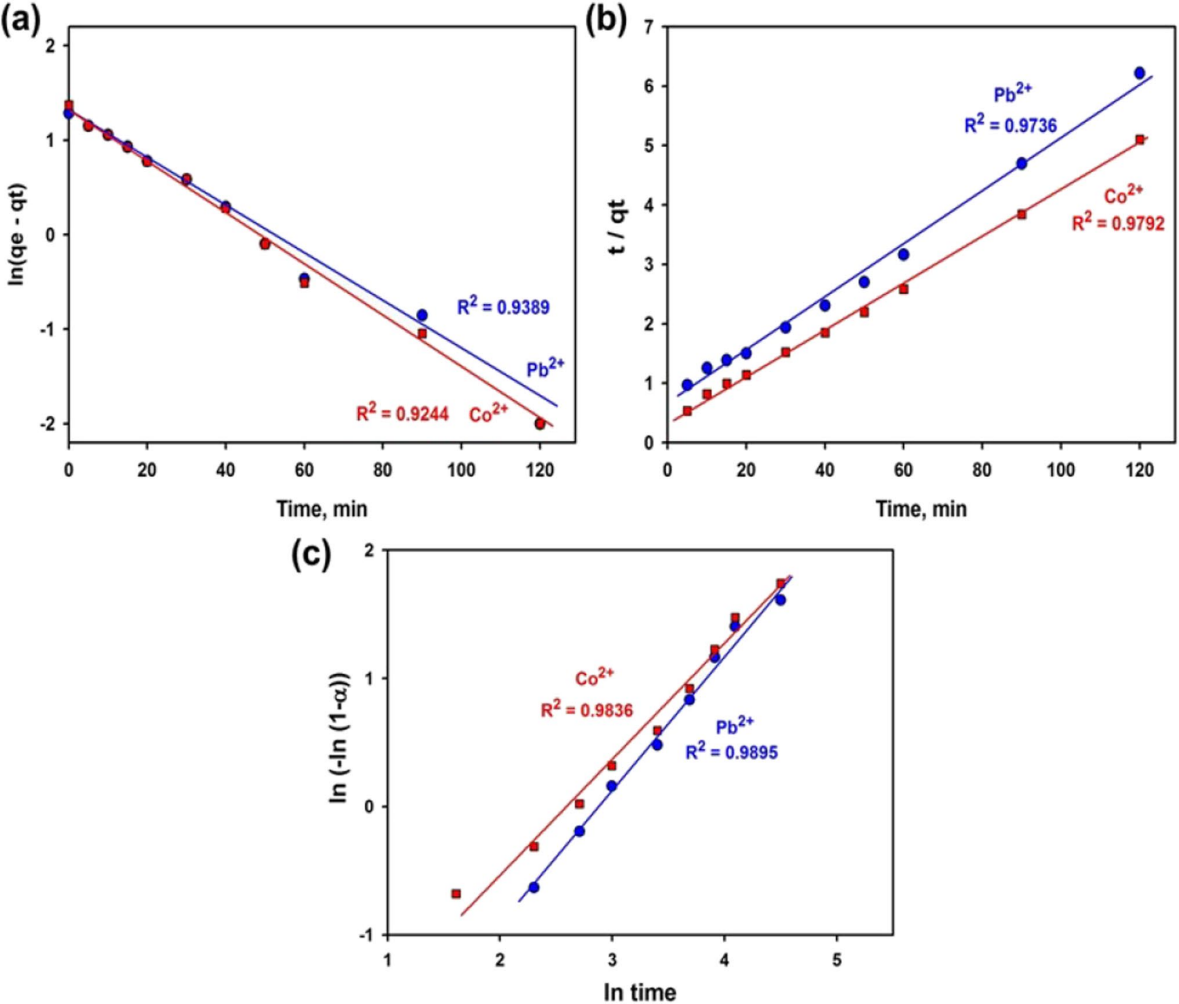
Plotting results according to the three kinetics models **a** pseudo-first-order, **b** pseudo-second-order, and **c** Avrami

The acquired outcomes of the three linearized models are displayed in Fig. 8; besides, their equivalent limits are listed in Table 4. Even though the PFO and PSO models are the most widely applied that forecast closer values of the equilibrium uptake capacity, the best-fitting was found using Avrami’s linear retrogression, based on *R*^2^ closest value to unity and low root mean square error (RMSE: 1.4782–2.7775). The kinetic fitting quality changes in the subsequent order: Avrami > PSO > PFO. The Avrami model has value of *R*^2^ (> 0.98).

**Table 4:** Different parameters of the kinetics models

Model	Item	Pb^2+^	Co^2+^
1^st^ order	*R*^2^	0.9389	0.9244
	*K*_1_	− 0.0614	− 0.0637
	*q*_max_ cal	20.4	21.6
	*q*_max_ exp	19.3	23.5
	RMSE	6.5114	8.3991
2^nd^ order	*R*^2^	0.9736	0.9792
	*K*_2_	0.0032	0.0043
	*q*_max_ cal	22.2	25.7
	*q*_max_ exp	19.3	23.5
	RMSE	4.5028	4.1203
Avrami	*R*^2^	0.9895	0.9836
	*n*	1.1797	1.1882
	*K*	0.0580	0.0759
	RMSE	1.4782	2.7775

*cal*. calculated, *exp*. experimental

Another kinetic model, reliant on chemical sorption, is the particle diffusion model. It entails the replacing or sharing electrons between the sorbent and metal ions (Jiang et al. 2010). It is assumed that adsorption occurs due to the flow of metal ions from the liquid to the sorbent exterior surface, ensued by ions dispersal into the pores. It is a time-consuming process that is proportional to time^0.5^ denoted as (Covelo et al. 2007):

13 $${\mathrm{q}}_{\mathrm{t}}={\mathrm{k}}_{\mathrm{id}}{\mathrm{t}}^{0.5}+\mathrm{I}$$

q_t_ is the amount of adsorbed ions after contact time t where t^0.5^ is its square root and K_id_ (mg g^−1^ min^−0.5^) is the rate constant and I is the intercept whose values provide statistics about the boundary layer’s depth, i.e., the bigger the intercept the larger the influence of that borderline layer is (Chouchane et al. 2021).

**Fig. 9:**
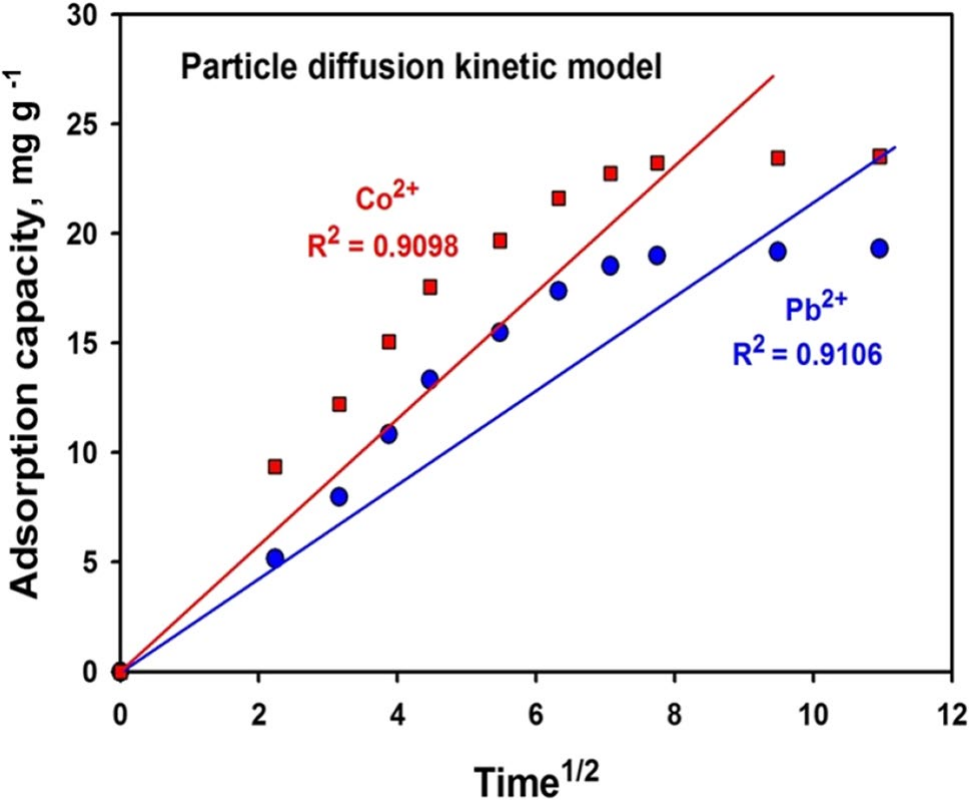
Plot of particle diffusion kinetic model

Figure 9 and Table 5 show that the diffusion represents a restraining phase in the operations on the sorbent. The increased adsorption capacity confirms the presence of mesopores, with a significant amount of sites, unlocked for the small ions. However, the linear plot of t^0.5^ against q_t_, fit data with good linear regression coefficients (*R*^2^ ≈ 0.91). It indicates the applicability of the model and the rate-monitoring step is intra-particle diffusion (Goldberg 2018).

**Table 5:** Parameters of particle diffusion model

Parameter	Pb^2+^	Co^2+^
linear regression coefficients (*R*^2^)	0.9106	0.9098
Thickness of boundary layer (I)	2.8206	5.4130
Rate constant of intra-particle diffusion (*K*_id_)	1.8917	2.1189

#### Temperature impact and sorption thermodynamics

**Fig. 10:**
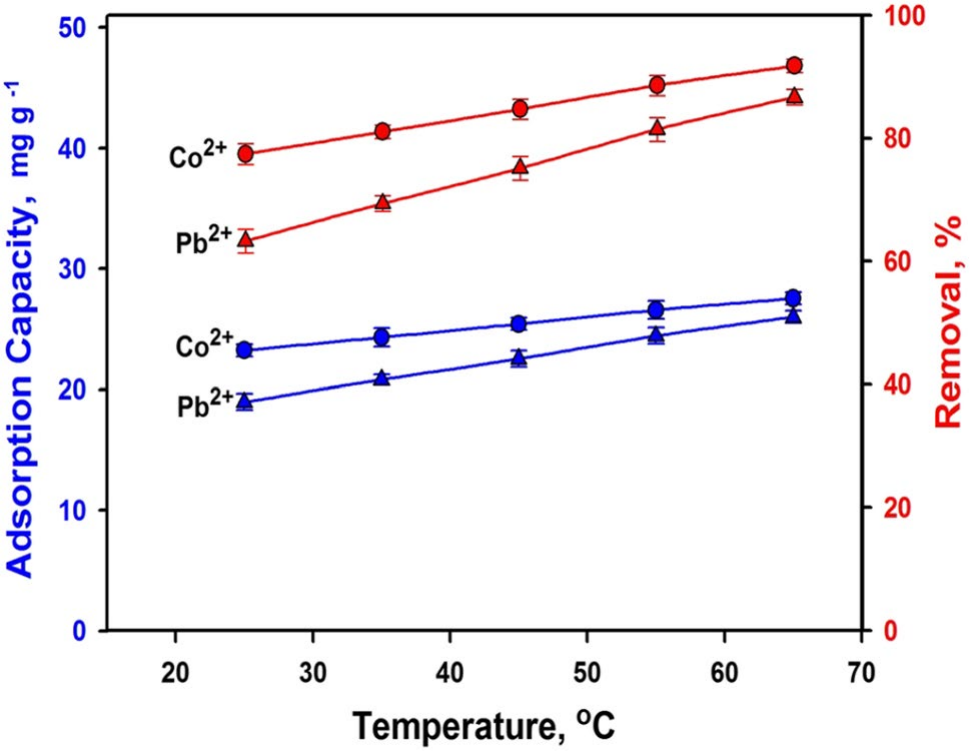
Effect of temperature on the removal efficiency of Co^2+^ and Pb^2+^ ions and adsorption capacity of BFS

Figure 10 displays the influence of temperature on the adsorption process. The extreme uptake capacity was reached at 65 °C, signifying that the adsorption is an endothermic process. Most adsorption studies suggest that increasing the temperature improves the sorption process (Argun 2008; Mercado-Borrayo et al. 2020; Plaza et al. 2021). Typically, at higher temperatures, the uptake is greater due to an increase in the energetic spots of the sorbent material. At increased temperatures, the system’s energy promotes the ions’ attachment to the mineral’s surface (Arief et al. 2008). Also, the movement of the ions becomes faster due to decreasing the viscosity of the solution (Fakari and Nezamzadeh-Ejhieh 2017), resulting in higher removal efficiencies (Rukayat et al. 2021).

**Fig. 11:**
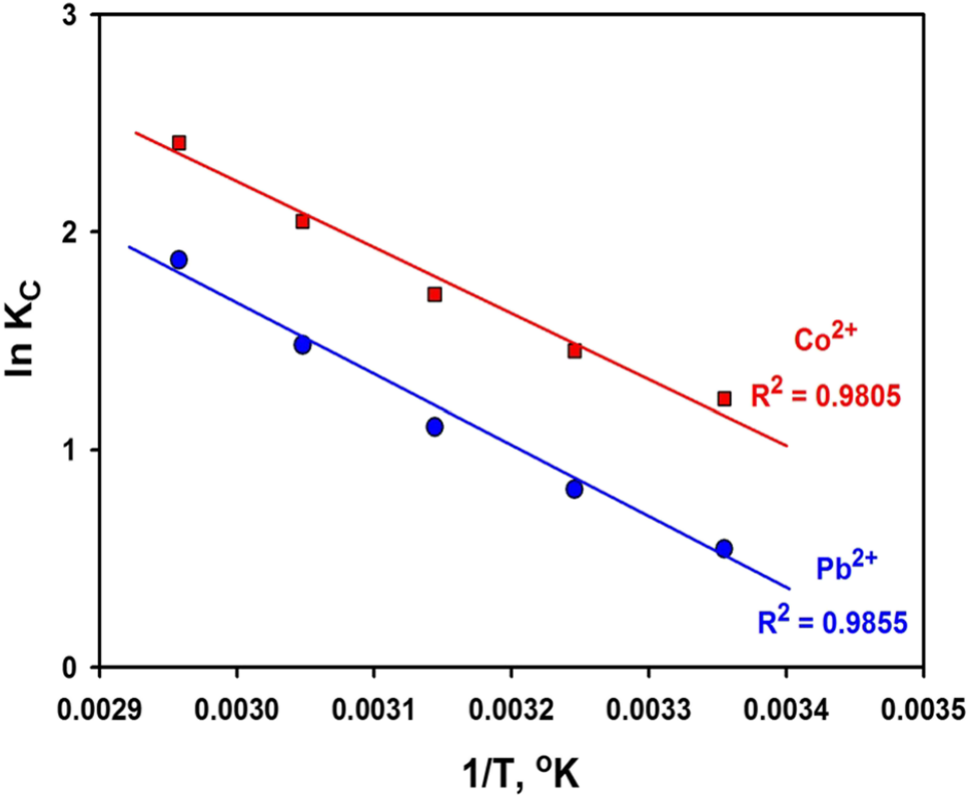
Plot of lnk_c_ versus 1/T

Changes in thermodynamics parameters such as Gibb’s free energy (ΔG°), entropy (ΔS°), and enthalpy (ΔH°) (Plaza et al. 2021) were determined. ΔH° and ΔS° were computed from Van’t Hoff equation (Karmaker et al. 2019):

14 $$\mathrm{ln}{K}_{c}=\frac{{\Delta S}^{^\circ }}{R}-\frac{{\Delta H}^{^\circ }}{RT}$$

where *kc* = *F*/(1 − *F*) and *F* = (*C*_0_ − *C*e)/*C*_0_ (Adeogun et al. 2018), *R* is the universal gas constant, and *T* is the temperature in K. The relationship of ln *kc* versus 1/T (Fig. 11) gives a straight line with a slope of − ΔH°/R and an intercept equals to ΔS°/R. The positive values of ΔH° in Table 6 specified the endothermic sorption process. Additionally, the ΔH° value < 30 kJ mol^−1^ verifies the physisorption process as shown by the Freundlich isotherm (Karmaker et al. 2019). Additionally, ΔS° was found to have positive values, suggesting a degree of unpredictability at the interface between the sorbent and adsorbate, inferring that sorption is less advantageous at lower temperatures.

**Table 6:** Thermodynamic parameters of Co^2+^ and Pb^2+^ ions sorption on BFS

Metal ion	Temp. (°C)	ΔG° (kJ mol^−1^)	ΔH° (kJ mol^−1^)	ΔS° (J mol^−1^ k^−1^)
(Pb^2+^)	25 35 45 55 65	− 1346 − 2092 − 2918 − 4032 − 5253	27.6	96.7
(Co^2+^)	25 35 45 55 65	− 3052 − 3719 − 4528 − 5586 − 6772	24.6	92.2

ΔG° is estimated via the subsequent relation (Hoang et al. 2019):

15 $${\Delta G^\circ =-RT\:lnK}_{c}$$

Table 6 shows that the sorption is unprompted since the ΔG° has negative values. It should be pointed out here that as the temperature rose, so did the negative values of ΔG°, suggesting that sorption is more favorable at higher temperatures (Ghasemi et al. 2020; Hassan et al. 2020).

#### Adsorption mechanism

As revealed by isotherm studies, the adsorption process fits the Freundlich isotherm thus obeying multilayer sorption of Pb^2+^ and Co^2+^ on BFS (physical adsorption). Inner layer sorption of metal ions on BFS might be ascribed to the creation of metal-Si complex between the Pb^2+^/Co^2+^ ion and Si of the slag via the exchange of H^+^ ions in the circumference. Moreover, the negatively charged BFS surface (at pH 6) favors the electrostatic interaction with positive metal ions.

As a result, the electrostatic interaction between Pb^2+^/Co^2+^ ions and the groups (such as − CO_3_ and − OH) on the sorbent is linked with multilayer adsorption of metal ions. Metal-sulfur complex formation via ion exchange and electrostatic interactions is thought to be a viable mechanistic mechanism for metal-ion adsorption on the BFS (Deng et al. 2020). Considering the nature and composition of the BFS, an exchange interaction of the slag with the effluent may be described as coming (Dimitrova and Mehandgiev 1998):

16 $$-\mathrm{SiO }\left(\mathrm{Ca}\right)+2\mathrm{H}-\mathrm{OH}\to -\mathrm{Si}-\mathrm{O}-{\mathrm{H}}_{2}+{\mathrm{Ca}}^{2+}+2\left(-{\mathrm{OH}}^{-}\right)$$

It can be anticipated that because of the large concentration of hydrogen ions in an acidic environment, the above reaction should shift to the left side. Following the aforesaid approach, the basic slags had a neutralizing impact.

Undoubtedly, Ca^2+^ ions interacted with the freed H^+^ ions from the slag when the solution pH rose, confirming the occurrence of the reaction in Eq. (17) when the BFS came in contact with solutions. The BFS slag exhibited a strong ion exchange capacity, which was consistent with the sorption equilibrium. For divalent metal ions (M^2+^) in solutions, the aforementioned equation may be expressed as (Zhan et al. 2019):

17 $${\left(\mathrm{Si}-{\mathrm{O}}^{-}\right)}_{2}{\mathrm{Ca}}^{2+}+{\mathrm{H}}_{3}{\mathrm{O}}^{+}\to 2\left(\mathrm{Si}-\mathrm{OH}\right)+{\mathrm{Ca}}^{2+}+{\mathrm{OH}}^{-}$$

**Fig. 12:**
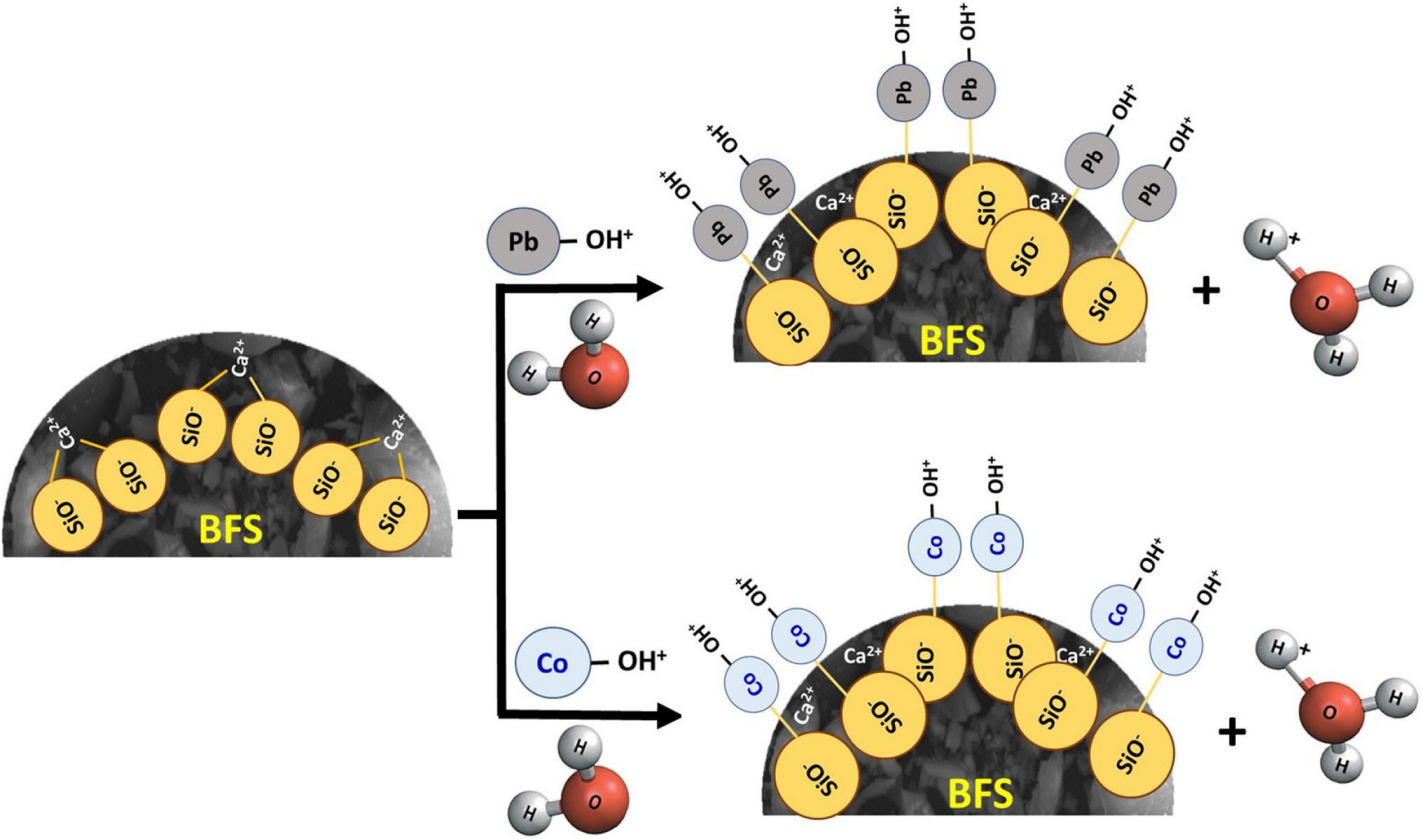
Schematic illustration of Pb and Co metal ions adsorption process by BFS

The lone pair of electrons in the oxygen atoms of OH^−^ groups play an important role in the complexation between metal ions and these OH^−^ groups (Wang et al. 2021) as illustrated in the schematic diagram shown in Fig. 12.

#### Regeneration and desorption

The regeneration and reusability of the slag for Co^2+^ and Pb^2+^ ions removal was examined under the maximum adsorption conditions: 50 mg L^−1^ initial ions concentration; 3.3 g L^−1^ slag dose; pH 6; at 65 °C; and time of 60 min. Whereas the conditions for regeneration were 0.1 M HNO_3_; solid/liquid ratio of 1:10; at 60 °C for 30 min contact. As revealed in Fig. S1, the adsorbed cobalt and lead ions on the slag surface could be efficaciously desorbed with efficiency exceeding 91% for the first cycle. Additionally, the removal efficiency was reduced by a few percent in the next three cycles. These results revealed that the slag could be reused repetitively to get rid of Co^2+^ and Pb^2+^ ions from discharge effluents.

It is worth noting that many laws in Egypt control wastewater reuse such as Egypt decrees no. 92/2013, and no. 208/2018 for protection of Nile River and its waterways from pollution coming from industrial activities (Egypt Decree 92/2013, Egypt decree No. 208/2018. Characteristics of the treated effluent in our study were complying with the permissible Egyptian limits for Co(II) (0.5 mg/L), Pb(II) (0.01 mg/L), and Fe (0.3 mg/L)for reclaimed water reuse standard according to these decrees.

The slag market is likely to vary, but industry efforts to promote “sustainable” materials and methods, as well as recycling in general, are likely to favor increased slag use and cost reduction. Furthermore, no chemicals were used in this study to convert BFS to adsorbent. Comparison with the other adsorbents such as activated alumina (0.60–1.19 × 10^3^ USD/ton), modified graphene oxide (⁓ 60 × 10^4^ USD/ton), and zeolite (30–120 × 10^3^ USD/ton) (Plaza et al. 2021), the cost of BFS will surely be much lower. The versatility of the described method is also enhanced by the fact that after the treatment, a settling process can easily separate the adsorbent from the effluent, allowing the adsorbent to be reused due to its high density.

To sum up, the present study using blast furnace slag has touched on the technical merit of slags that can be used as efficient adsorbents for decontaminating waste effluents of industries. Additionally, the gained outcomes assert promisingly that the considered process may meet the requirements of using slag adsorbents at a cheap and plentiful source for large-scale production. Comparison with the other iron steel and slag adsorbents used for Pb^2+^ and Co^2+^ removal from wastewater is exhibited in Table S3 in the supplemental file.

## Conclusion

Blast furnace slag (BFS) has a complex composition. Its surface charge is pH-dependent, where the isoelectric point is at pH 4.8. It was successfully used, without modification, as a sorbent of heavy metal ions. Its selectivity follows the order of Co^2+^  > Pb^2+^ which is attributed to the hydration energy and charge density. The Freundlich isotherm model fits well indicating the physical nature of the sorption progression and the dimensionless separation factor (R_L_) indicates its favorability. The higher adsorption amount in the first duration verified the sorption favorability on the BFS exterior surface over that in the interior pores. The particle diffusion model describes that the sorption is occurred by the ions flowing from the aqueous phase to the sorbent’s exterior surface, pursued by ions dispersion into the apertures and pores. Kinetic studies carried out indicated that the Avrami kinetic model best described the adsorption mechanism. Furthermore, the thermodynamic parameters revealed that the adsorption process was endothermic and spontaneous in nature. The slag could be regenerated and reused repetitively to remove Pb^2+^ and Co^2+^ ions from effluents. In conclusion, BFS represents a comparatively effective, low-cost, and environmentally friendly adsorbent that can be applied industrially in the field of wastewater pollution control for the purpose of Pb^2+^ and Co^2+^ ions removal.

## Supplementary Information

Below is the link to the electronic supplementary material.

Supplementary file1 (DOCX 219 KB)

## Data Availability

The authors confirm that all data supporting the study’s findings are included in the article.
