# Supplementary material for: From waste to waste: iron blast furnace slag for heavy metal ions removal from aqueous system
Source: Environ Sci Pollut Res Int. 2022 Mar 31;29(38):57964–79. doi: 10.1007/s11356-022-19834-3 (PMC9395503; doi:10.1007/s11356-022-19834-3)
Supplement: Supplementary file 1 — Supplementary file1 (DOCX 219 KB) [file 11356_2022_19834_MOESM1_ESM.docx]

**Supporting information**

**From waste to waste: Iron blast furnace slag for heavy metal ions removal from aqueous system**

Sabah M. Abdelbasir^*^, M.A. Abdel Khalek

*Central Metallurgical Research and Development Institute, P.O. Box: 87, Helwan, 11421, Cairo, Egypt*

*^*^Corresponding author. Tel.: +202 27142011; fax: +202 27142451.*

*E-mail address:* [*sfoda20@hotmail.com*](mailto:sfoda20@hotmail.com)

ORCID/ 0000-0003-2698-2041

**Slag characterization**

Chemical composition of BFS was determined using X-ray Fluorescence (XRF, Axios Advanced WDXRFP analytical, Netherland). The detection limit for each element reported as metal oxides. The mineral composition was investigated using X-ray diffraction (Bruker D8 Advance diffractometer, Germany), with Cu-Kὰ radiation (40 kV, 40 mA) (λ = 1.5406 Å in a 2θ range of 10 - 70°). Microscopic structural investigation of BFS components was performed by field emission scanning electron microscope (FESEM, QUANTA FEG 250 manufacturers, Holland) with an **EDX unit** (JEOL, JSM-4510, Germany).

The surface area was determined by the nitrogen adsorption technique (Brunauer-Emmett-Teller (BET) isotherm) at 77 K using a Quantachrome instrument (Nova Series, UK) of degassed samples at 100°C for 5h. A thermostatic shaker model (GFL3017, Germany) was used for shaking during experimentations with a constant rate. The adsorbents were separated using a centrifuge (Sigma, Germany) at a speed of 8000 rpm to achieve complete separation.

**Table S1.** Surface structure of BFS under N_2_

| **Specific surface area (m^2^ g^-1^)** | **pore volume**  **(cc/g)** | **average pore size**  **(nm)** |
| --- | --- | --- |
| 19.315 | 0.018 | 3.144 |

**Table S2.** Physical and chemical properties of the studied metals (Nightingale 1959) (Volkov et al. 1997)

| **Element** | **Atomic mass**  **(g/mol)** | **Atomic radius**  **(Å)** | **Ionic radius**  **(Å)** | **Hydration Energy**  **(KJ mol^–1^)** | **Charge density*** | **Hydrated ionic radius**  **(Å)** | **Solubility product constants of metal hydroxide (25°C)** |
| --- | --- | --- | --- | --- | --- | --- | --- |
| Co | 58.93 | 1.67 | 0.65 | –1996 | 2.7778 | 2.10 | 1.3 ×10^-15^ |
| Pb | 207.20 | 1.81 | 1.32 | –1480 | 1.6667 | 4.01 | 1.43×10^-20^ |

* Charge density = charge / ionic radius


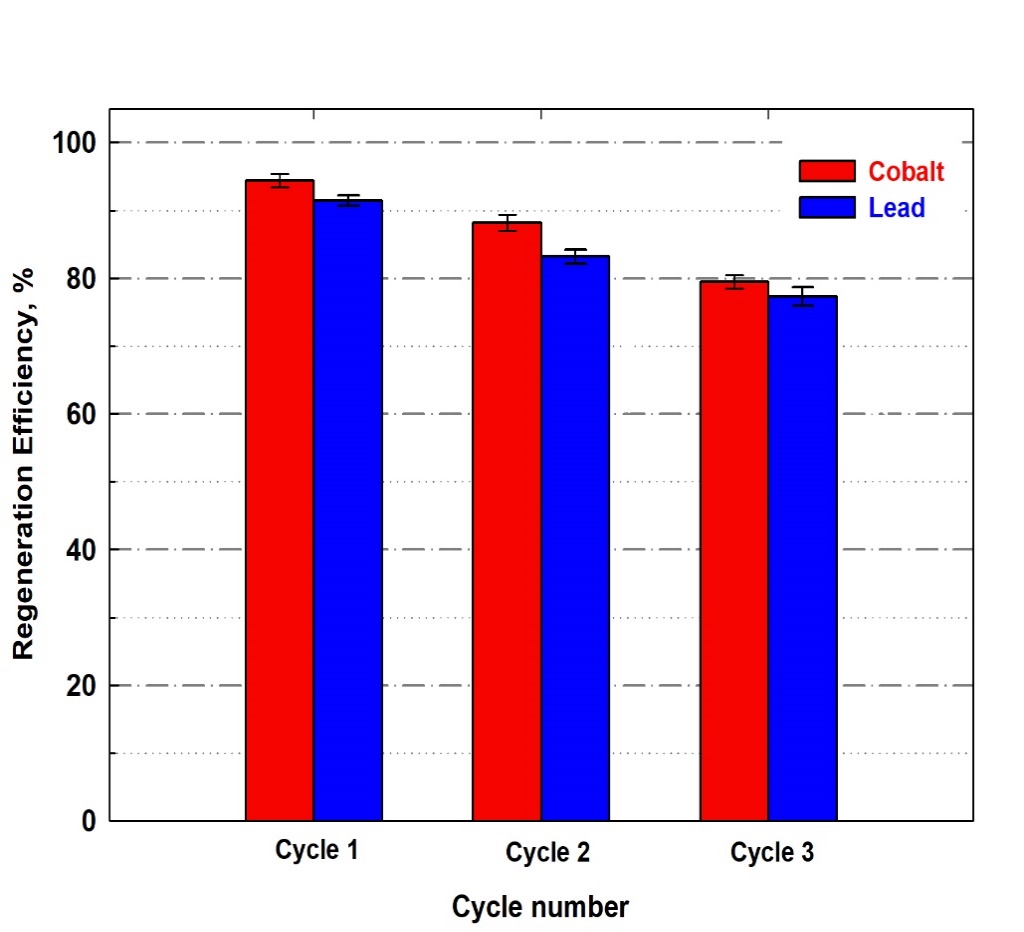


**Fig. S1.** Reusability performance of BFS for Co^2+^ and Pb^2+^ ions removal.

**Table S3.** Comparison of Pb^2+^ and Co^2+^ removal from wastewater using different slag adsorbents.

| **Adsorbent** | **Adsorption capacity (mg g^−1^) or % Removal** | | **Adsorption isotherm** | **Kinetic model** | **References** |
| --- | --- | --- | --- | --- | --- |
|  | Pb^2+^ | Co^2+^ |  |  |  |
| Steel slag | 32.3 |  | Langmuir | ^*^NR | (Feng et al. 2004) |
| Iron slag | 95.2 |  | Langmuir | ^*^NR | (Feng et al. 2004) |
| Iron oxide-coated sludge | 42.4 |  | Langmuir | PSO | (Phuengprasop et al. 2011) |
| Oxalated blast-furnace slag |  | 576 | Sips | PSO | (Le et al. 2021) |
| Granulated blast-furnace slag | 97% |  | Freundlich | ^*^NR | (Dimitrova and Mehandgiev 1998) |
| Bentonite-Steel Slag Composite | 29.8 |  | Langmuir and Brunauer Emmett Teller (BET) | PSO | (Zhan et al. 2019) |
| Basic oxygen furnace slag (BOFs) | 98.3 |  | Freundlich | PSO | (Xue et al. 2020) |
| Electric induction furnace slag | 34% |  | ^*^NR | ^*^NR | (Mercado-Borrayo et al. 2020) |
| Blast furnace slag (non-modified) | 30.2 | 43.8 | Freundlich | Avrami | ***This work*** |

^*^NR: not reported by the author.

**Supporting references**

Dimitrova S v., Mehandgiev DR (1998) Lead removal from aqueous solutions by granulated blast-furnace slag. Water Research 32:3289–3292. https://doi.org/10.1016/S0043-1354(98)00119-5

Feng D, van Deventer JSJ, Aldrich C (2004) Removal of pollutants from acid mine wastewater using metallurgical by-product slags. Separation and Purification Technology 40:61–67. https://doi.org/10.1016/J.SEPPUR.2004.01.003

Le QTN, Vivas EL, Cho K (2021) Oxalated blast-furnace slag for the removal of Cobalt(II) ions from aqueous solutions. Journal of Industrial and Engineering Chemistry 95:57–65. https://doi.org/10.1016/J.JIEC.2020.12.003

Mercado-Borrayo BM, Contreras R, Sánchez A, et al (2020) Optimisation of the removal conditions for heavy metals from water: A comparison between steel furnace slag and CeO2 nanoparticles. Arabian Journal of Chemistry 13:1712–1719. https://doi.org/10.1016/j.arabjc.2018.01.008

Nightingale ER (1959) Phenomenological Theory of Ion Solvation. Effective Radii of Hydrated Ions. The Journal of physical chemistry 63:1381–1387. https://doi.org/10.1021/J150579A011

Phuengprasop T, Sittiwong J, Unob F (2011) Removal of heavy metal ions by iron oxide coated sewage sludge. Journal of hazardous materials 186:502–507. https://doi.org/10.1016/J.JHAZMAT.2010.11.065

Volkov AG, Paula S, Deamer DW (1997) Two mechanisms of permeation of small neutral molecules and hydrated ions across phospholipid bilayers. Bioelectrochemistry and Bioenergetics 42:153–160. https://doi.org/10.1016/S0302-4598(96)05097-0

Xue YJ, Hu ZH, Niu YY (2020) Single and coadsorption of copper, cadmium, lead and zinc onto basic oxygen furnace slag. Desalination and Water Treatment 179:242–251. https://doi.org/10.5004/DWT.2020.24868

Zhan X, Xiao L, Liang B (2019) Removal of Pb(II) from Acid Mine Drainage with Bentonite-Steel Slag Composite Particles. Sustainability 2019, Vol 11, Page 4476 11:4476. https://doi.org/10.3390/SU11164476
